# Intuitive teaching of medical device operation to clinical assistance robots

**DOI:** 10.1007/s11548-022-02802-0

**Published:** 2022-12-09

**Authors:** Oskar Baumann, Alexander Lenz, Josef Hartl, Lukas Bernhard, Alois Christian Knoll

**Affiliations:** 1 Chair of Robotics, Artificial Intelligence and Real-time Systems, Technical University of Munich, Boltzmannstr. 3, 85748 Garching, Germany; 2 Research Group MITI, Klinikum rechts der Isar der Technischen Universitt Mnchen, Trogerstr. 26, 81675 Munich, Germany

**Keywords:** Clinical assistance systems, Intuitive robots, Learning from demonstration, Expert systems, Medical device adjustment

## Abstract

**Purpose:**

The adjustment of medical devices in the operating room is currently done by the circulating nurses. As digital interfaces for the devices are not foreseeable in the near future and to incorporate legacy devices, the robotic operation of medical devices is an open topic.

**Methods:**

We propose a teleoperated learning from demonstration process to acquire the high-level device functionality with given motion primitives. The proposed system is validated using an insufflator as an exemplary medical device.

**Results:**

At the beginning of the proposed learning period, the teacher annotates the user interface to obtain the outline of the medical device. During the demonstrated interactions, the system observes the state change of the device to generalize logical rules describing the internal functionality. The combination of the internal logics with the interface annotations enable the robotic system to adjust the medical device autonomously. To interact with the device, a robotic manipulator with a finger-like end-effector is used while relying on haptic feedback from torque sensors.

**Conclusion:**

The proposed approach is a first step towards teaching a robotic system to operate medical devices. We aim at validating the system in an extensive user study with clinical personnel. The logical rule generalization and the logical rule inference based on computer vision methods will be focused in the future.

## Purpose

In today’s healthcare system, the shortage of qualified personnel and the resulting overload of healthcare workers pose major challenges [1]. One approach to ease the situation is the integration of *autonomous self-navigating clinical assistance systems* (ASCAS) into the existing workflows [2]. These robotic systems can alleviate the workload of healthcare workers by performing monotonous or physically strenuous tasks, allowing the human personnel to focus on tasks of direct patient care.

A mobile service robot currently developed in the scope of the research project *Autonomous Self-Navigating Robotic Operating Room Assistance* (AURORA) is transferring these concepts into the operating room itself by automating selected tasks that are currently performed by circulating nurses. Next to the handling of sterile goods, the adjustment of medical devices is one of the main tasks during operation. As the development of digital interfaces to control these medical devices is slowed down by strict regulation and security concerns, the robotic system is required to use the mechanical, human-centreed interfaces of the medical devices. Using this approach, legacy devices without digital interfaces can be incorporated as well.

Due to the amount of different devices, it is desirable that the robot learns the device functionality and does not depend on hard-coded solutions by technical experts. To speed up the learning process and to ensure proper functionality in a safety-critical environment, the knowledge of the medical staff can be utilized by having them supervise the learning process as a “teacher.” While the interfaces of medical devices are built with simplicity in mind, the challenge of teaching a robot to operate the devices is still non-trivial.

This short communication introduces a technical setup and workflow where a human operator teaches the operation of medical devices to the robot by interacting with a digital twin of the device presented in a graphical user interface (GUI). The robot mirrors the actions taken by the user on the virtual representation and performs them on the physical device. This realizes a remote teaching process where the clinical personnel can teach the robot intuitively without having to be present in the operating room.

## Methods

The clinical environment poses specific challenges on the learning from demonstration (LfD) problem. As the environment is time-critical and the equipment is also not available for extensive training sessions, the learning has to be fast, and subsequently only a limited amount of data is available to train on. Additionally, in the operating room, requirements on safety are high as the patient outcome cannot be endangered. Therefore, many more recent LfD methods, which focus on learning motion primitives alongside of high-level plans [3], are not fit for the problem. Instead, we propose to use an expert system based on *Prolog* [4] to encode the internal functionality of the device, which provides an interpretable and explainable model.

For the teleoperated teaching process, we use a similar approach to [5] where the elements were annotated on a tablet before experimenting with the operation of the interaction elements. To localize the devices, we are using *ArUco* markers [6] to determine which devices are in sight. Afterwards, to obtain the exact pose, we use the scale-invariant feature transform [7] to match the features of the detected device interface with the camera image. We use the *ssocr* [8] library to evaluate seven-segment displays.

**Fig. 1:**
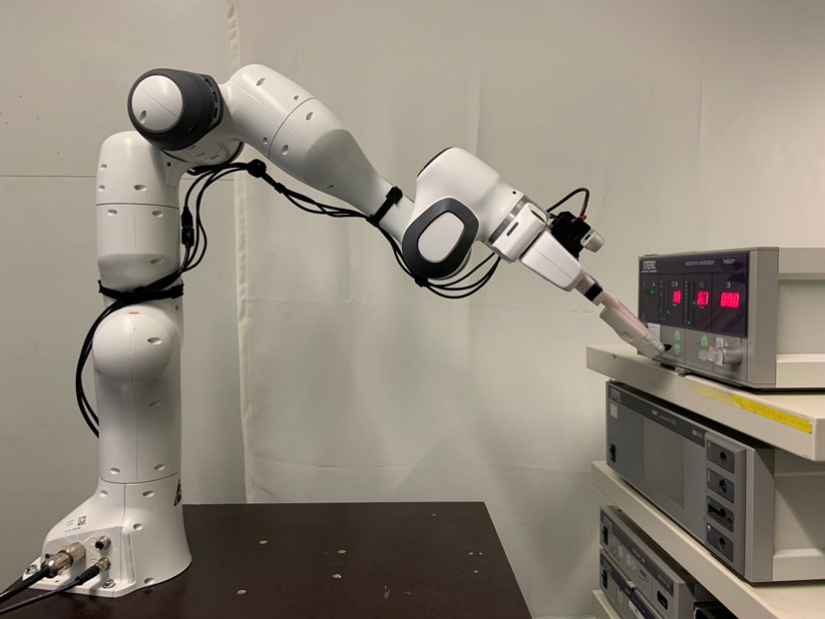
Setup consists of the seven degree of freedom, collaborative robot arm PANDA of FRANKA EMIKA [9] and an insufflator [10] on a medical equipment cart

**Fig. 2:**
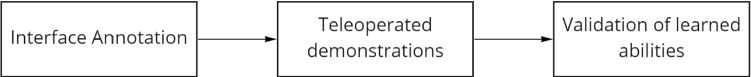
Visualization of the learning process

The project AURORA aims at developing a mobile service robot. The mobile platform is not relevant for the isolated task of the device adjustment and therefore is ignored in this paper. The robotic setup is shown in Fig. 1. The robot arm PANDA of FRANKA EMIKA [9] is mounted on top of a static platform. The end-effector consist of a camera and a manipulator to actuate the medical device. The RGBD camera [11] is used to read the medical devices and perform collision avoidance during robot arm movements. The manipulator is a fixed finger-like structure without any additional sensors or actuators. To validate the methods, an insufflator [10] is used as an exemplary medical device. The interface of this insufflator consists of several push buttons and a rocker to switch on the device. The device displays the current state using three seven-segment displays and several bar graphs and LEDs.

## Results

The resulting system is discussed by starting with the teaching process from the user’s perspective, before the technical details are explained. The device representation is discussed first. Afterwards, the robotic interaction with user interfaces and the logic engine is outlined.

### Teaching process

**Fig. 3:**
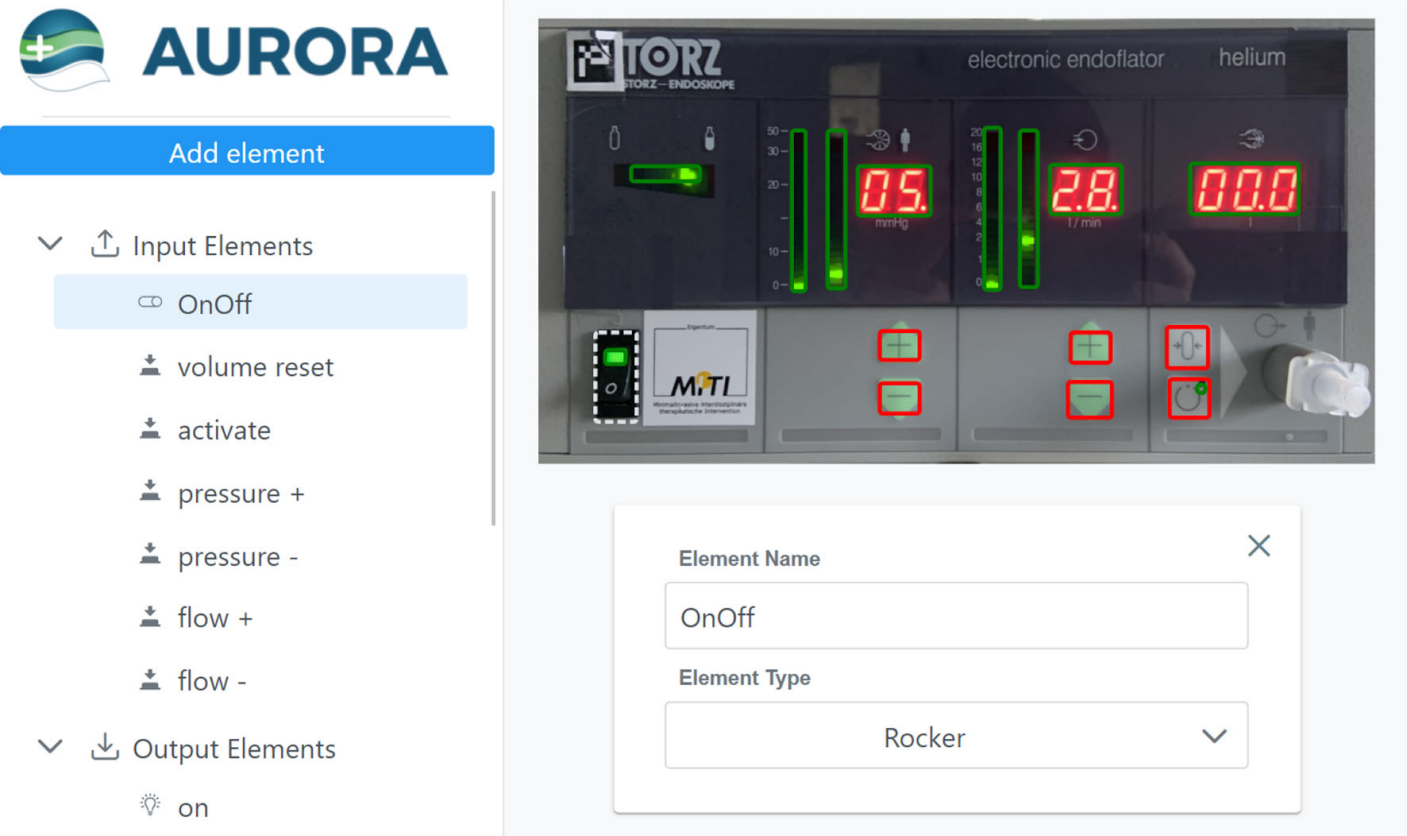
Annotation software while annotating the insufflator. The elements of the interface are listed on the left, while the interface is visible on the right. The rocker button to switch the device on is selected

The complete teaching process (Fig. 2) can be done remotely with a tablet displaying the GUI. The process is initiated when the robot detects an unknown device. The first step of the teaching procedure is the annotation of the user interface to obtain the external description of the user interface, also called the affordances of the device. In the beginning, the user marks the outer bounds of the interface in the camera image. The robot then moves to centre the device interface in the camera frame. The image of the device interface is saved alongside the affordances in order to localize the device. Thereafter, the interaction elements in the interface are marked and annotated with a button type and a human-readable name (Fig. 3).

After completing the annotation, the user demonstrates the usage of the device. The user interacts with a digital twin of the device interface, which leads to a sequence of commands that the robot performs on the actual device at the same time. The output state of the device is mirrored back to the digital representation such that the user can see the current state. During this process, the user gets feedback on the coverage percentage of the elements to derive the overall teaching progress.

To conclude the teaching, the user tests the ability of the robot to operate the device by choosing a goal state of the device and checking if the robot is able to reach it. If an error occurs, the systems falls back into the demonstration phase and requires the teacher to add more knowledge. This test is repeated until the human teacher decides that the device is completely understood by the robot.

### Device representation

**Fig. 4:**
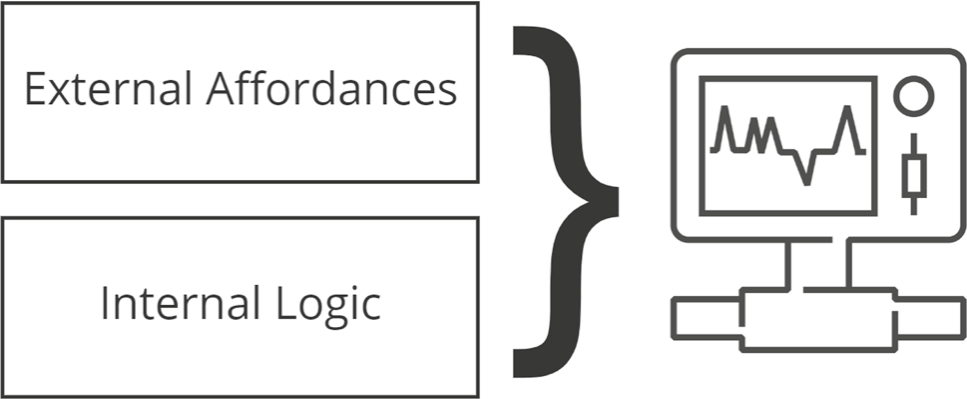
Device description is separated into the external description of the physical interface (affordances) and the internal description of the device in form of the logic

The device description is separated into the description of external elements and internal logic (Fig. 4). The external description, also referred to as affordances, details the structure of the physical interface of the device and contains all interaction elements. The internal logic contains the functionality of the device and describes how the interaction with the physical elements affect the internal state of the device.

The affordances of the device are the collection of all interaction elements. Each element is defined by a unique name, the relative position inside the device interface and an element type. These types are grouped into input and output elements. In [5], an extensive study on robotic actuation of buttons and switches was conducted, and a categorization of those elements by physical properties was introduced. Those categories pose the subset of input elements. The output elements consist of LEDs, seven-segment displays and bar graphs.

The internal functionality of the device is represented by logical clauses describing under which precondition the interaction with an element on the interface leads to a certain outcome. A custom clause *action* is defined to represent the logic of the device (Listing 1).
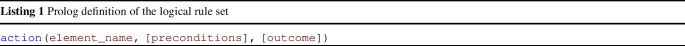

**Fig. 5:**
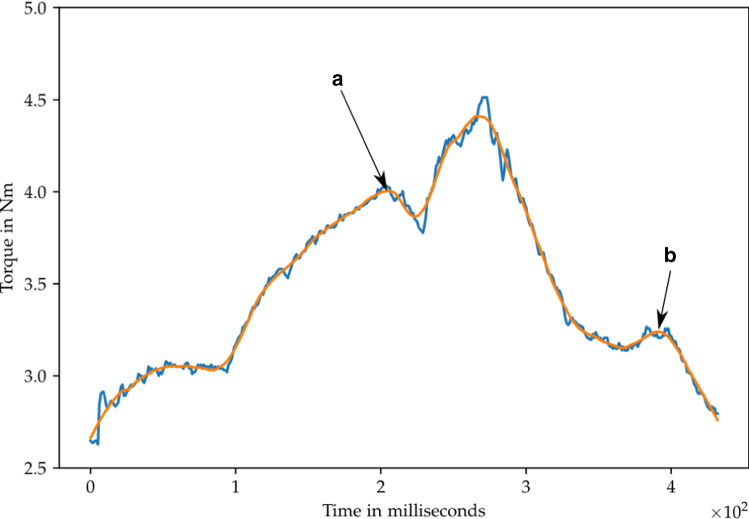
Sensor readings from the torque sensor in the last joint during a single button pressing. The blue line contains the raw data, while the orange line displays filtered sensor data. Point a and b mark the moments when the button is pressed and released, respectively

### Robot device interaction

The interaction with the device utilized fixed routines that can operate the elements if given the relative position of the element in the interface and the element type. By not trying to learn the control of the robotic arm itself, the learning process is faster and also the safety of the movement can be ensured as the depth data generated by the camera are used to avoid collision during all movements.

During the interaction with the medical devices, the torque sensors of the robot arm are utilized to realize haptic feedback. The haptic feedback allows the robot to detect the tactile point where the force has reached the necessary level to press the button and the button is activated. By detecting the tactile point, it is possible to detect whether a button was indeed successfully pressed. In order to keep the moving mass low during the operation of the device, the robot first approaches a position with the end-effector placed right on the element before moving only the last joint to actuate the element, which is similar to a human button operation [12]. To calculate the force on the element, the torque of the last joint is used together with a lever length of 0.44 m, which leads to better readings than estimating the 3D contact force using multiple joints torques and the Jacobian. The corresponding torque measurements are shown in Fig. 5 where the measured torque during a single rotational button pressing is displayed. Additionally, the tactile points are marked.

### Logic engine

The experience gained from interacting with the physical device contains all interactions with prior state and resulting state. The experience set allows interpolating the device operation and to infer action sequences in already known regions. In order to also extrapolate the device behaviour, it is necessary to derive a generalized logical rule set from existing experiences. With each new interaction, the gained experience is saved and checked against the generalized rules to see if there is a conflict which requires a new computation of the generalized rules. In Listing 2 exemplary experience data are displayed for increasing the pressure. The corresponding generalized rules can be seen in Listing 3.
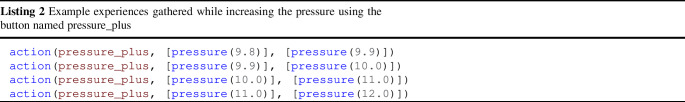

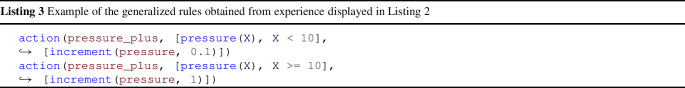


After the teaching process is concluded, the logical rules are used to query an action sequence to reach a desired goal state from the current state. The rules are used to perform a *Breadth-first search (BFS)*. Each rule is tested whether the preconditions are met and if so, the resulting state and action sequence is added to the search tree until the goal state is reached.

## Discussion and conclusion

In this paper, we proposed a workflow enabling clinicians to teach a robot to operate medical devices. A GUI allows the non-technical user to annotate unknown devices and to demonstrate the device operation remotely. The affordances are used to operate the device with haptic feedback and to generate a digital twin to command a desired device state in a GUI. The logic engine allows the system to extrapolate the device behaviour outside the demonstrated regions. By encoding the behaviour into sets of logical rules, the system is insensitive against faulty or suboptimal demonstrations. However, incomplete demonstrations can lead to faulty adjustments in unknown regions. In case of failure of the logical rules in a real application, the GUI can also be used to teleoperate the robot and ensure a fast and convenient error recovery. Currently, the setup is limited to two-dimensional user interfaces but is extensible to arbitrary interface shapes by 3D scanning the device and altering the user-interface to work on a 3D model instead of a 2D image. Future work will focus on the effective rule generalization and the derivation of rules based on the interface configuration. A further open field is the computer vision-based annotation of the device interface to ease the workload of the human operator. The output elements need to be extended by units in order to allow a natural language interaction that uses the affordances to interpret the intent of the speaker and retrieve the mentioned goal state. Additionally, the results have to be validated by extensive user studies.
